# Unraveling the Contribution of *MulSOS2* in Conferring Salinity Tolerance in Mulberry (*Morus atropurpurea* Roxb)

**DOI:** 10.3390/ijms25073628

**Published:** 2024-03-24

**Authors:** Hai-Rui Wang, Sheng-Mei Han, Dong-Hao Wang, Zhen-Rui Zhao, Hui Ling, Yun-Na Yu, Zhao-Yang Liu, Ying-Ping Gai, Xian-Ling Ji

**Affiliations:** 1 College of Forestry, Shandong Agricultural University, Taian 271018, China; 18678320797@163.com (H.-R.W.); hanshengmei203@163.com (S.-M.H.); wangdh1204@163.com (D.-H.W.); lzysdau@163.com (Z.-Y.L.); 2 College of Life Sciences, Shandong Agricultural University, Taian 271018, China; 18364758328@163.com (Z.-R.Z.); 15866118045@163.com (H.L.); yunnayu99@163.com (Y.-N.Y.)

**Keywords:** mulberry, SOS2, salt stress, transgenic plant, salt tolerance

## Abstract

Salinity is one of the most serious threats to sustainable agriculture. The Salt Overly Sensitive (SOS) signaling pathway plays an important role in salinity tolerance in plants, and the *SOS2* gene plays a critical role in this pathway. Mulberry not only has important economic value but also is an important ecological tree species; however, the roles of the *SOS2* gene associated with salt stress have not been reported in mulberry. To gain insight into the response of mulberry to salt stress, *SOS2* (designated *MulSOS2*) was cloned from mulberry (*Morus atropurpurea* Roxb), and sequence analysis of the amino acids of MulSOS2 showed that it shares some conserved domains with its homologs from other plant species. Our data showed that the *MulSOS2* gene was expressed at different levels in different tissues of mulberry, and its expression was induced substantially not only by NaCl but also by ABA. In addition, *MulSOS2* was exogenously expressed in Arabidopsis, and the results showed that under salt stress, transgenic *MulSOS2* plants accumulated more proline and less malondialdehyde than the wild-type plants and exhibited increased tolerance to salt stress. Moreover, the *MulSOS2* gene was transiently overexpressed in mulberry leaves and stably overexpressed in the hairy roots, and similar results were obtained for resistance to salt stress in transgenic mulberry plants. Taken together, the results of this study are helpful to further explore the function of the *MulSOS2* gene, which provides a valuable gene for the genetic breeding of salt tolerance in mulberry.

## 1. Introduction

Soil salinization is one of the important environmental factors limiting land use and crop production [1]. It is estimated that about one fifth of the world’s irrigated land is affected by salinization, and by the middle of the 21st century, more than 50 percent of the land will be salinized, which has attracted extensive attention from the international community [2]. The increase in salt concentration in soil will lead to salt stress, which will damage plant root tip cells, affect the absorption and transfer of water and nutrients by roots, and hinder the normal growth of plant roots [3]. Soil salinization not only affects the growth and development of plants but also easily leads to a series of secondary ecological environment crises, seriously restricting the sustainable development of agriculture and forestry and the stability of ecosystem. Plant salt tolerance is a complex quantitative trait controlled by multiple genes, involving multiple metabolic pathways and signaling pathways [4]. It is of great significance to explore plant salt-tolerant genes and plant salt-tolerant mechanisms for improving plant salt tolerance and crop yield, breeding new salt-tolerant varieties, and promoting the development and utilization of saline–alkali land [5].

Mulberry has a long history of cultivation in China and is the important material basis for the sericulture industry [6]. It not only has important economic value, but also is an important ecological tree species that plays an important role in windbreak and sand fixation, soil and water conservation and saline–alkali land management [7]. In recent years, with the development of animal husbandry, the reduction in grassland resources, and the intensification of the food crisis, mulberry is not only used as silkworm food but also used as food for cattle, sheep, pigs, and other animals, showing broad prospect for development and utilization. At present, the sericulture industry in China is gradually shifting from the eastern to the western inland arid and salinized areas. Therefore, exploring salt-tolerant genes of mulberry and breeding salt-tolerant varieties are of great significance for improving the utilization efficiency of saline–alkali land and promoting the sustainable development of the sericulture industry.

When plants are subjected to salt stress, they can receive external salt stress signals through different receptors, then convert them into intracellular signals and transmit them through the plant via complex signal cascade pathways, and then regulate gene expression at the overall level, thereby reestablishing cellular ion, osmotic, and reoxidation equilibrium homeostasis to adapt to salt stress and enhance plant salt tolerance [8]. Previous studies have shown that the Salt Overly Sensitive (SOS) signal transduction pathway is an important signaling pathway in the response to salt stress in plants [9], and it is a Ca^2+^ dependent signal transduction pathway which is prevalent and highly conserved in higher plants. The SOS pathway is mainly composed of SOS1, SOS2, SOS3, and SOS3-like proteins (SCaBP8) [10,11,12]. Among them, SOS1 is a Na^+^/H^+^ antiporter, which can exclude excessive Na^+^ accumulated in the cytoplasm and reduce the toxic effect of Na^+^ on cells under salinity stress [13,14]. Moreover, it plays an important role in regulating the concentration of K^+^ and the homeostasis of pH in vacuoles, as well as enhancing the salt tolerance of plants [15,16]. The *SOS2* gene encodes a serine/threonine (Ser/Thr) protein kinase with a catalytic region similar to the yeast sucrose non-fermenting 1 (SNF1) belonging to the SnRK3 subfamily [17]. It has been shown that SOS2 has an N-terminal kinase catalytic domain similar to SNF1/AMPK, and its C-terminal contains a FISL domain, which is the self-inhibiting region of the protein. SOS3 is an EF hand calcium-binding protein which is myristoylated at its N terminus and serves as a calcium sensor in plants [18]. Under salt stress, plants can sense salt stress through extracellular salt sensors and increase the intracellular Ca^2+^ level. Then, SOS3 and SCaBP8 can transduce the Ca^2+^ signal to the SOS2 protein by recruiting it to the cell membrane and reduce the phosphorylation level of SOS2 [19]. Thus, the inhibition of 14-3-3 protein on SOS2 is alleviated, and SOS2 is activated [20,21]. Subsequently, the activated SOS2 phosphorylates and activates SOS1, which extrudes excess Na^+^ out of the cell, thus conferring salt tolerance in plants [22]. Therefore, SOS2 is considered to be the central factor in the SOS signaling pathway and plays a key role in plant salt tolerance [23]. It has been reported that in addition to being involved in the response to salt stress, the *SOS2* gene can also be induced by abscisic acid (ABA) and various abiotic stresses and may have a variety of physiological functions [24]. So far, the *SOS2* gene has been functionally characterized in a variety of plant species, and it has been shown that overexpression of *SOS2* can improve the salt tolerance of different transgenic plants [25,26,27]. Unfortunately, research on the function of the *SOS2* gene in mulberry is still lacking. At the same time, due to the lack of an efficient genetic transformation and regeneration system in mulberry, many genes that have been proven to have salt tolerance in other plants have not been validated in mulberry trees. So far, there have been no reports of transgenic salt tolerance genes in mulberry trees.

To explore the role and mechanism of *MulSOS2* in salt tolerance in mulberry, the *MulSOS2* gene was cloned and molecularly characterized, and the expression profiles of the gene were analyzed. Meanwhile, the functions of *MulSOS2* in regulating plant salt tolerance were explored. These findings will lay the groundwork for further exploring the role and mechanism of *MulSOS2* in salt tolerance and may provide effective genetic resources for improving mulberry resistance to salt tress.

## 2. Results

### 2.1. Isolation of MulSOS2 from Mulberry and Analysis of Its Deduced Protein

**Figure 1:**
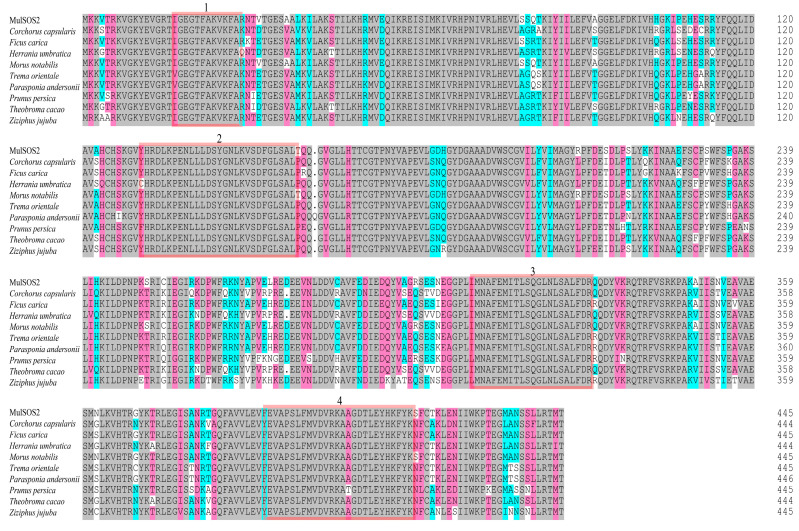
Multiple alignment of amino acid sequences of SOS2 proteins from different plants. Identical amino acid residues are shown in gray shadows, and similar amino acids are shown in red shadows. The red boxes 1–4 indicate the protein kinase ATP binding region, serine/threonine kinase active site, protein kinase domain, and calcium/calmodulin-dependent protein kinases, respectively. The proteins used to be aligned were from *Corchorus capsularis* (OMO81155.1), *Ficus carica* (GMN58210.1), *Herrania umbratica* (XP 021277487.1), *Morus notabilis* (EXB21296.1), *Trema orientale* (PON89026.1), *Parasponia andersonii* (PON63234.1), *Prunus persica* (XP 007202049.1), *Theobroma cacao* (XP 007013470.2), and *Ziziphus jujuba* (XP 048337577.2).

**Figure 2:**
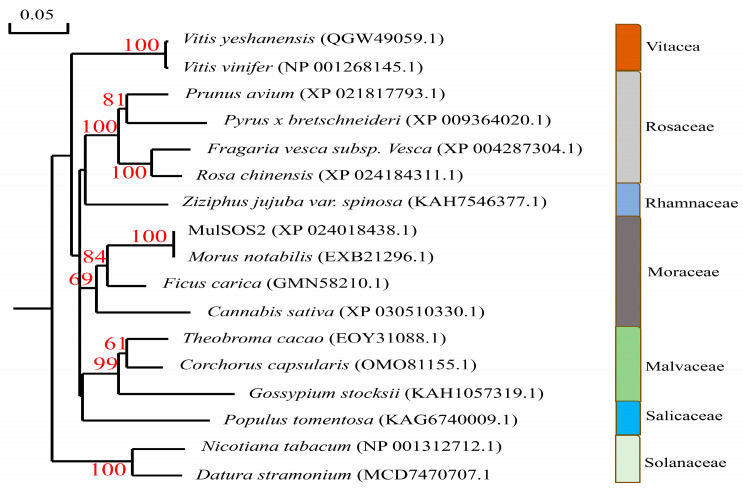
Phylogenetic analysis of MulSOS2 protein and its homologous proteins. GenBank accession numbers of the selected proteins are shown in the parentheses. Phylogenetic tree was constructed by the neighbor-joining method with 1000 bootstraps. The scale indicates genetic distance, and the bootstraps are given beside the branches.

*MulSOS2* cDNA was isolated from the leaves of Guisang You 62 (*Morus atropurpurea* Roxb), and it encoded a protein containing 446 amino acids with a molecular weight of 50.24 kDa and an isoelectric point of 8.66. The online software Plant-mPLoc (http://www.csbio.sjtu.edu.cn/bioinf/plant-multi/) (accessed on 5 January 2024) was used to analyze the subcellular localization of the MulSOS2 protein, and the results showed that it was localized in the cytoplasm. Moreover, the amino acid sequence of MulSOS2 was alimented with those of SOS2 proteins of other species, and the results showed that they have some high conserved domains, such as protein kinase ATP binding sites, serine/threonine protein kinase active sites, a protein kinase domain, and a calcium/calmodulin-dependent protein kinase domain. However, the amino acid sequences in other regions were less conserved (Figure 1). In addition, the phylogenetic tree of the MulSOS2 protein and SOS2 proteins of other species was constructed, and the results showed that it has the closest homology to the SOS2 protein of *Morus notabilis*, while it has a distant phylogenetic relationship with the SOS2 proteins of *Nicotiana tabacum* and *Vitis yeshanensis* (Figure 2).

### 2.2. Expression Characteristics of MulSOS2 Gene

**Figure 3:**
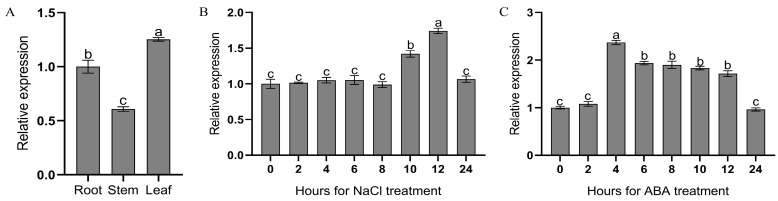
Expression pattern of the *MulSOS2* gene. (**A**–**C**) indicate the tissue expression characteristics, salt-induced expression characteristics, and ABA-induced expression characteristics of the *MulSOS2* gene, respectively. For ABA treatment, five-week-old mulberry seedlings were sprayed with 100 μmol∙L^−1^ of ABA. As for NaCl treatment, five-week-old mulberry seedlings were irrigated with 200 mmol∙L^−1^ NaCl solution. The relative expression levels were evaluated using the comparative Ct method with *EF1-α* and *Actin* as reference genes. Data represent the average of triplicate samples ± SD. The columns marked with different letters above represent significant differences (*p* < 0.05) according to Duncan’s multiple range test.

Firstly, the expression levels of the *MulSOS2* gene in the roots, stems, and leaves of five-week-old mulberry seedlings were analyzed by qRT-PCR, and the results showed that *MulSOS2* was expressed in the roots, stems, and leaves of mulberry, but its expression levels were significantly different in different tissues, with the highest expression level in the leaf and the lowest expression level in the stem (Figure 3A). Moreover, the mulberry seedlings were treated with 200 mmol∙L^−1^ NaCl solution, and the expression characteristics of *MulSOS2* under salt stress were investigated. The results showed that its expression was significantly induced by salt stress and reached the maximum expression level 12 h after salt treatment. This suggests that the *MulSOS2* gene may play an important role in the response to salt stress in mulberry (Figure 3B). In addition, the mulberry seedlings were treated with 100 μmol∙L^−1^ ABA, and the induced expression characteristics of *MulSOS2* were explored by qRT-PCR analysis. The results showed that the expression level of *MulSOS2* was significantly increased 4 h after ABA treatment and remained at a high level from 6 to 12 h after treatment, until it returned to the pre-treatment level 24 h after treatment (Figure 3C). Therefore, the expression of *MulSOS2* may also be associated with ABA.

### 2.3. Ectopic Expression of MulSOS2 in Arabidopsis Enhances Salt Tolerance of the Transgenic Plants

**Figure 4:**
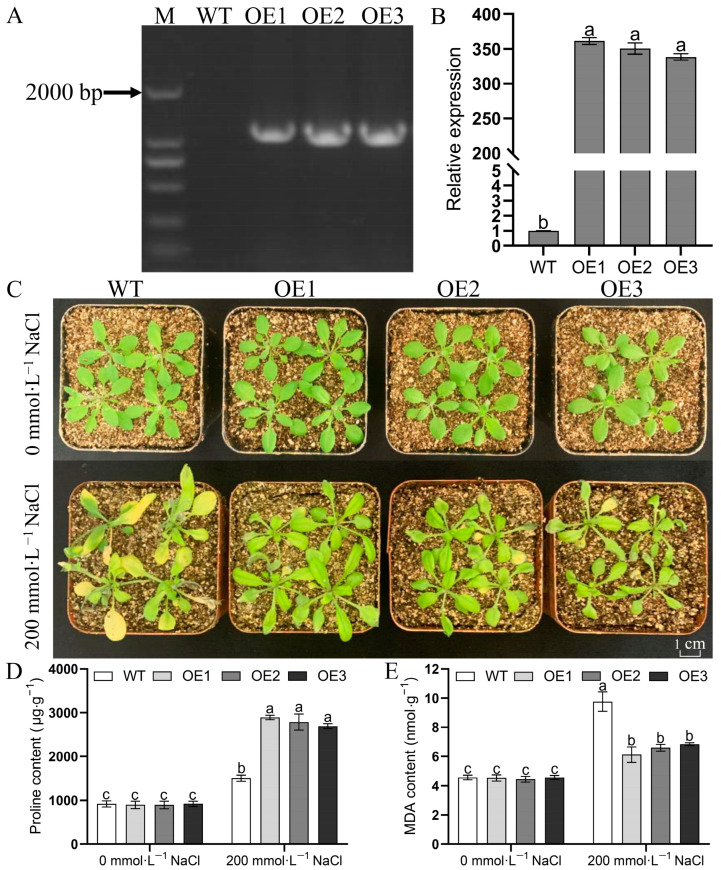
Phenotypic and physiological characteristics of the transgenic *MulSOS2* Arabidopsis treated with NaCl. (**A**): PCR identifications of the transgenic *MulSOS2* gene Arabidopsis plants. (**B**): Expression levels of *MulSOS2* in transgenic plants. (**C**): Effects of salt stress on the morphology of transgenic *MulSOS2* Arabidopsis seedlings. (**D**,**E**) indicate proline and malondialdehyde (MDA) contents in Arabidopsis seedlings, respectively. Arabidopsis seedlings were treated with 200 m mol∙L^−1^ NaCl for 48 h, and the leaves were collected for proline and MDA content measurement. M: DNA Marker 2000; WT: wild-type plant; OE1–OE3: different transgenic *MulSOS2* lines. Data represent the mean values of three biological replicates ± standard deviation, and different letters above the columns indicate significant differences (*p* < 0.05) according to Duncan’s multiple range test.

To analyze the role of *MulSOS2* in response to salt stress, transgenic *MulSOS2* Arabidopsis plants were generated. Genome PCR analysis results showed that *MulSOS2* was successfully integrated into the Arabidopsis genome (Figure 4A), and qRT-PCR analysis results indicated that *MulSOS2* was successfully expressed in the transgenic Arabidopsis seedlings (Figure 4B). Then, the four-week-old wild type (WT) and overexpression (OE) *MulSOS2* Arabidopsis seedlings were treated with 200 mmol·L^−1^ NaCl solution, respectively. Seven days after treatment, the phenotypes of Arabidopsis plants were observed, and the results showed that the WT plants showed more severe symptoms of salt damage, such as leaf yellowing and wilting, and the growth of the plants was significantly inhibited. Although the leaves of OE seedlings also showed yellowing and wilting, the symptoms of salt damage were less than those of WT plants, and the growth of the plants was not significantly inhibited (Figure 4C). Moreover, the contents of proline and malondialdehyde (MDA) in the leaves of WT and OE plants under salt stress were also measured. The results showed that under salt treatments, the proline content was lower but the MDA level was significantly higher in the WT seedlings compared to those in the OE ones (Figure 4D,E). These results indicated that the ectopic expression of *MulSOS2* in Arabidopsis enhances the salt tolerance of transgenic plants.

**Figure 5:**
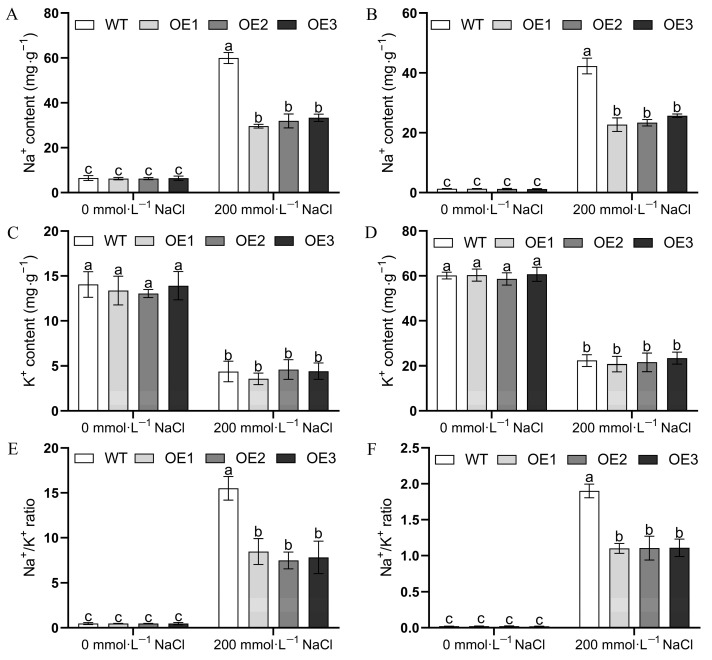
Na^+^ and K^+^ content in the roots and leaves of transgenic plants and wild-type Arabidopsis plants. (**A**,**B**) show the Na^+^ contents in the roots and leaves, respectively. (**C**,**D**) show the K^+^ contents in the roots and leaves, respectively. (**E**,**F**) show the Na^+^/K^+^ ratios in the roots and leaves, respectively. WT: wild-type plant; OE1–OE3: different transgenic *MulSOS2* lines. Data represent the mean values of three biological replicates ± standard deviation, and different letters above the columns indicate significant differences (*p* < 0.05) according to Duncan’s multiple range test.

To explore whether the overexpression of *MulSOS2* can reduce Na^+^ accumulation in transgenic *MulSOS2* Arabidopsis seedlings, the Na^+^ and K^+^ contents in the leaves and roots of the transgenic and WT plants were measured. The results showed that there was no significant difference in Na^+^ and K^+^ contents in the leaves and roots between transgenic and WT plants without NaCl treatment. Though the Na^+^ contents in the leaves and roots of transgenic and WT seedlings were all increased under 200 mmol∙L^−1^ NaCl treatment, the leaves and roots of WT plants accumulated more Na^+^ than those of transgenic *MulSOS2* plants (Figure 5A,B). Under NaCl treatment, the K^+^ contents in the leaves and roots of transgenic and wild-type plants were all decreased, and there was no significant difference in K^+^ contents in the leaves and roots between the transgenic and wild-type plants (Figure 5C,D), but the Na^+^/K^+^ ratios in the leaves and roots of wild-type plants were higher than those of transgenic plants (Figure 5E,F).

### 2.4. Overexpression of MulSOS2 in Mulberry Enhances Salt Tolerance

**Figure 6:**
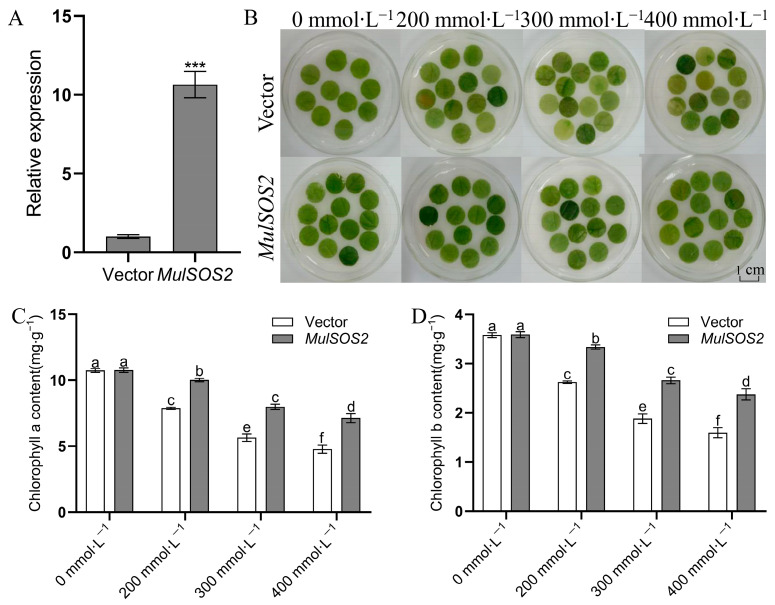
Salt tolerance of mulberry leaves with the transient overexpressed *MulSOS2* gene. (**A**): Expression level of the *MulSOS2* gene in mulberry leaves; (**B**): Phenotypes of mulberry leaves upon NaCl solution treatment. (**C**,**D**) show the chlorophyll a and b contents, respectively. Vectors: transgenic empty-vector leaf discs which were used as controls; *35S::MulSOS2*: transgenic *MulSOS2* leaf discs. Data represent the mean values of three biological replicates ± standard deviation, *** above the columns indicates significant differences (*p* < 0.001) according to *t* tests, and different letters above the columns indicate significant differences (*p* < 0.05) according to Duncan’s multiple range test.

To further verify the salt tolerance function of the *MulSOS2* gene, it was transiently overexpressed in mulberry leaves, and the expression levels of *MulSOS2* in the transient infected leaves were analyzed by qRT-PCR. The results showed that the expression levels of *MulSOS2* in the transgenic *MulSOS2* leaves were significantly higher than those in the transgenic empty-vector ones, indicating the *MulSOS2* gene was overexpressed in the transgenic *MulSOS2* leaves (Figure 6A). Then, leaf discs of 1 cm diameter were prepared from the leaves and floated in 0 mmol·L^−1^, 200 mmol·L^−1^, 300 mmol·L^−1^, and 400 mmol·L^−1^ NaCl solutions, respectively. Four days after treatment, the phenotype and chlorophyll contents of the discs were examined. Under the control (0 mmol·L^−1^ NaCl) condition, there was no significant difference between transgenic empty-vector and transgenic *MulSOS2* leaf discs. But under NaCl stress (200 mmol·L^−1^, 300 mmol·L^−1^ and 400 mmol·L^−1^ NaCl), the leaf discs from transgenic *MulSOS2* leaves showed more tolerance to stress (Figure 6B). At the same time, the chlorophyll content measurement results showed that the chlorophyll a and b contents of the transgenic *MulSOS2* leaf discs were significantly higher than those of the transgenic empty-vector ones (Figure 6C,D). These results indicate that the overexpression of the *MulSOS2* gene in mulberry leaves can improve the tolerance of the leaves to salt stress.

**Figure 7:**
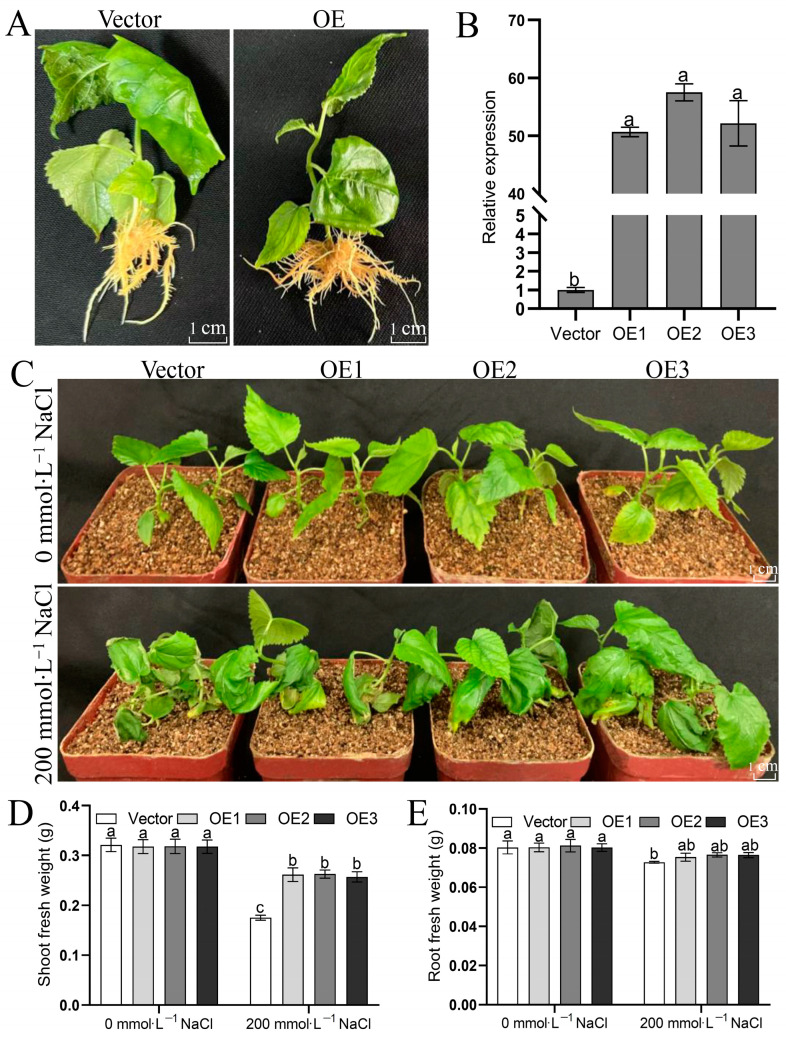
Salt tolerance of mulberry seedlings carrying transgenic *MulSOS2* hairy roots. (**A**): Mulberry seedlings carrying hairy roots. (**B**): Expression levels of the *MulSOS2* gene in the mulberry hairy roots. (**C**): Phenotypes of mulberry seedlings carrying hairy roots upon 200 mmol·L^−1^ NaCl solution treatment. (**D**,**E**) show the shoot and root fresh weights of the seedlings, respectively. Data represent the mean values of three biological replicates ± standard deviation, and different letters above the columns indicate significant differences (*p* < 0.05) according to Duncan’s multiple range test. Vectors: mulberry seedlings carrying transgenic empty-vector hairy roots, which were used as controls; OE1–OE3: different lines of mulberry seedlings carrying transgenic *MulSOS2* hairy roots.

Furthermore, transgenic *MulSOS2* and transgenic empty-vector hairy roots were obtained (Figure 7A). qRT-PCR analysis showed that the expression level of *MulSOS2* in the transgenic *MulSOS2* hairy roots was significantly higher than that in transgenic empty-vector hairy roots, indicating that *MulSOS2* gene was successfully overexpressed in the transgenic *MulSOS2* hairy roots (Figure 7B). Then, the mulberry seedlings carrying transgenic *MulSOS2* or transgenic empty-vector hairy roots were treated with 200 mmol·L^−1^ NaCl solution, respectively. Seven days after treatment, it was found that the mulberry seedlings carrying transgenic empty-vector hairy roots showed more severe symptoms of salt damage, all the leaves were wilted, and the lower leaves were even withered. However, the mulberry seedlings carrying transgenic *MulSOS2* hairy roots showed mild symptoms of salt damage, the leaves were less wilted, and the top leaves remained a normal green color (Figure 7C). Moreover, it was shown that there was no significant difference in the shoot and root fresh weight between the mulberry seedlings carrying transgenic *MulSOS2* hairy roots and empty-vector hairy roots (Figure 7D,E). However, after treatments with 200 mmol·L^−1^ NaCl, the mulberry seedlings carrying transgenic empty-vector hairy roots exhibited significantly lower fresh shoot weight than the mulberry seedlings carrying transgenic *MulSOS2* hairy roots (Figure 7D). In addition, it was shown that after treatments with 200 mmol·L^−1^ NaCl, the fresh root weights of the seedlings carrying transgenic empty-vector hairy roots were lower than those treated with 0 mmol·L^−1^ NaCl, while there was no significant difference in the fresh root weight of the seedlings carrying transgenic *MulSOS2* hairy roots between the seedlings treated with 0 mmol·L^−1^ NaCl and those treated with 200 mmol·L^−1^ NaCl (Figure 7E). These results indicate that *MulSOS2* gene overexpression in the hairy roots can significantly improve the salt tolerance of the mulberry seedlings. Therefore, the *MulSOS2* gene plays an important role in salt tolerance in mulberry.

## 3. Discussion

It was proposed that *SOS2* is a potential candidate gene for enhancing salt tolerance in plants, and isolating the *SOS2* gene is of great significance in plant salt tolerance breeding [28]. However, the function of SOS2 in enhancing salt tolerance in mulberry is yet unexplored. In this study, the *SOS2* gene of mulberry, designated as *MulSOS2*, was isolated and characterized. At present, the efficient genetic transformation and regeneration system of mulberry has not been established. To explore the function of *MulSOS2* in mulberry, it was transiently overexpressed in mulberry leaves and stably expressed in the hairy roots of mulberry. To the best of our knowledge, this is the first report on the function of *MulSOS2* in mulberry. Multiple alignment of the amino acid sequences showed that the MulSOS2 protein was highly homologous with the SOS2 proteins from other plants, especially in the protein kinase ATP binding sites, serine/threonine protein kinase active sites, protein kinase domain, and calcium/calmodulin-dependent protein kinase domain (Figure 1). Sequence conservation of these functional domains of MulSOS2 and other SOS2 proteins indicated that they may have similar functions and mechanisms in plant salt tolerance.

It has been reported that the expression of *SOS2* may be related to multiple factors, including salt stress, tissue, plant variety, and other factors [29,30]. In this study, the tissue-specific expression patterns of *MulSOS2* were analyzed, and the results showed that *MulSOS2* was expressed in the roots, stems, and leaves (Figure 3A). It is worth noting that the *MulSOS2* gene is highly expressed in the leaves, followed by the roots, indicating that it may have different biological functions in different tissues. Moreover, our data showed that the expression of the *MulSOS2* gene was significantly up-regulated under salt stress (Figure 3B). It has been reported that the *SOS2* gene can also be induced by ABA in addition to salt stress and may have various functions [24]. Our data also showed that the expression of the *MulSOS2* gene was induced by ABA (Figure 3C). Since ABA levels can be changed under multiple stresses, it was suggested that ABA might modulate the expression of *MulSOS2* in the response to a variety of stresses. Therefore, *MulSOS2* may have multiple stress-induced expression characteristics and play important roles in the response to various stresses.

Previous studies have shown that the SOS pathway plays an important role in regulating cellular ion homeostasis, and SOS2 is a key gene controlling plant salt tolerance [31,32,33]. It has been reported that SOS2 can regulate not only the activity of SOS1, which extrudes excess Na^+^ out of the cell, thus conferring salt tolerance in plants, but also the activity of cation/proton antitransporters, and transgenic plants overexpressing *SOS2* exhibit improved salt tolerance [22,24,34]. Our data showed that the Na^+^ contents in transgenic *MulSOS2* Arabidopsis plants were lower as compared to those in wild-type plants under salt treatment (Figure 5A,B). Moreover, it was found that the K^+^ contents in Arabidopsis plants were decreased under salt treatment, but the K^+^ contents in the leaves and roots of transgenic *MulSOS2* plants were higher than those in wild-type plants (Figure 5C,D). Therefore, overexpression of the *MulSOS2* gene may make transgenic plants decrease the accumulation of Na^+^ and maintain a low Na^+^/K^+^ ratio in the cell, and this may endow transgenic plants with stronger salt tolerance than wild-type plants. It was well known that high concentrations of Na^+^ will disrupt ion homeostasis in plant cells, resulting in osmotic stress and ionic toxicity to plants and inducing reactive oxygen species (ROS) accumulation [35,36,37,38]. Excessive accumulation of ROS may lead to oxidative stress, which may cause cell membrane degradation and lipid peroxidation, resulting in oxidative damage to plants [39]. SOS2 is considered to be one of the nodes connecting salt stress response and ROS signaling, linking the SOS pathway with superoxide metabolism [40]. Our data showed that under salt stress, MDA content in WT plants increased significantly more than that in transgenic *MulSOS2* plants (Figure 4E). This indicates that *MulSOS2* overexpression in transgenic plants is helpful to control the balance of cellular redox status and lipid peroxidation, reduce oxidative damage to the cell membrane, and enhance tolerance to salt stress. The accumulation of osmolytes is one of the physiological mechanisms by which plants respond to salt stress, and proline is an important osmoprotectant, which plays an important role in osmotic adjustment and elevating plant salt tolerance [41]. In our study, under salt treatment, the content of proline in transgenic *MulSOS2* plants was higher than that in WT plants (Figure 4D). Therefore, the overexpression of *MulSOS2* may enhance osmotic adjustment capacity in transgenic plants, which may partly explain why the overexpression of *MulSOS2* can improve salt stress tolerance. Further studies are required to elucidate the precise mechanism behind the role of *MulSOS2* in regulating plant salt tolerance.

## 4. Materials and Methods 

### 4.1. Biological Materials and Reagents

*Arabidopsis thaliana* (Col-0) seedlings were cultured in incubators at 22 °C with a humidity of 50–60% and a light/dark regime (16 h light(100 μmol·m^−2^·s^−1^ photon flux density)/8 h dark). Guisang You 62 (*Morus atropurpurea* Roxb) seedlings were cultured in a greenhouse at 25 °C with a humidity of 50–60% and a light/dark regime (16 h light (100 μmol·m^−2^·s^−1^ photon flux density)/8 h dark). The seedlings were fertilized twice weekly by sub-irrigation with 0.25 × Hoagland’s medium (pH 6.0).

The biochemical reagents we used were all purchased from Shanghai Biotechnology Co., LTD. (Shanghai, China), unless otherwise specified.

### 4.2. RNA Extraction, Gene Cloning and Sequence Analysis

For cDNA synthesis, mulberry leaves were used to isolate total RNAs using TRIzol^®^ RNA Isolation Reagent (Invitrogen, Carlsbad, CA, USA) following the manufacturer’s instructions, and the RNAs obtained were reverse-transcribed with M-MLV reverse transcriptase (Promega, Madison, WI, USA). Specific PCR primers (forward primer: 5′-TCTCTCTCTCCCTCTCTGC-3′; reverse primer: 5′-TAAGATACACCACGGGAG-3′) were designed and used for the amplification of *MulSOS2*, and the PCR amplification products were purified and sequenced. Amino acid sequence alignments of MulSOS2 with the SOS2 proteins from other plants were conducted using the DNAMAN program, and a phylogenetic tree was constructed with MEGA11 software (version 11.0.13) (accessed on 5 January 2024) by the neighbor-joining method with 1000 bootstrap replications. The web-based tool Plant-mPLoc (http://www.csbio.sjtu.edu.cn/bioinf/plant-multi/) (accessed on 5 January 2024) was used to predict the subcellular localization of the MulSOS2 protein.

### 4.3. Quantitative Real-Time PCR Analysis

Quantitative real-time PCR analyses were performed on a StepOnePlus™ Real-time PCR System (Applied Biosystems, Waltham, MA, USA) with specific primers (forward primer: TAGGCGATACTTTCAACAACT; reverse primer: TGTGTGAAGAAAGACCAACC) and the SYBR Premix Ex Taq™ kit (Takara Bio., Kusatsu, Shiga, Japan) according to the protocol provided by the kit. *ACTIN* (forward primer: 5′-GCACCCTGTTCTTCTTACCG-3′; reverse primer: 5′-AACCCTCGTAGATTGGCACA-3′) and *EF1-a* (forward primer: GGGTGATTCAAGATGATGACT; reverse primer: TCAGTCAAGGACATCCGAAG) were used as references, and the gene expression level of the *MulSOS2* gene was quantified by the comparative Ct method. Triplicate technical replicates were performed for all samples.

### 4.4. Production of Transgenic Arabidopsis

For Arabidopsis transformation, the coding region of *MulSOS2* was ligated into the expression vector pBI121 under the control of the CaMV 35S promoter and introduced into *Agrobacterium tumefaciens* strain GV3101. Meanwhile, the empty pBI121 vector was introduced into GV3101. *Agrobacterium* strains harboring individual vectors were used to transform Arabidopsis with the floral dip method [42]. The transgenic seeds were sowed and selected on kanamycin (50 μg·mL^−1^) selection medium, and T2 homozygous seeds selected were used for subsequent experiments.

### 4.5. Transformation of Mulberry Leaves and Production of Transgenic Hairy Roots

The vectors obtained above were transformed into *A. tumefaciens* strain GV3101, respectively. *Agrobacterium* stains harboring individual vectors were inoculated in LB media with appropriate antibiotics. An overnight culture of *Agrobacterium* was harvested at OD_600_ of 0.8, centrifuged at 5000× *g* for 5 min, and resuspended in 100 mL of infiltration medium (10 mmol∙L^−1^ MgCl_2_, 5 mmol∙L^−1^ MES-KOH (pH 5.6), and 200 μmol∙L^−1^ acetosyringon). The bacterial solution was incubated at room temperature for 3 h with gentle shaking under dark conditions. The third young leaves of five-week-old Guisang You 62 mulberry seedlings were soaked in *Agrobacterium* solution, and then *Agrobacterium* infiltration was performed by applying a vacuum three times for 5 min [43]. After infiltration, the leaf petioles were wrapped with absorbent cotton, and then the leaves were cultured in Petri dishes for 2 d for subsequent experiments.

As for the production of transgenic hairy roots, the coding region of MulSOS2 was subcloned into the pROK2 vector under the control of the CaMV 35S promoter [44]. Then, the created vector and empty pROK2 vector were introduced into A. rhizogenes K599 strains, respectively. Then, 200 μL of A. rhizogenes K599 strains were cultured in 20 mL of LB liquid medium (supplemented with 50 g∙L^−1^ kanamycin and 50 g∙L^−1^ streptomycin) for 12 h with shaking at 200 r∙min^−1^, and then the culture was centrifuged at 6000× *g* for 5 min. The A. rhizogenes K599 strains were collected and resuspended in the solution (2.132 g∙L^−1^ 2-N-morpholino ethanesulfonic acid, 2.033 g∙L^−1^ MgCl_2_∙6H_2_O, 200 μmol∙L^−1^ acetosyringone, pH = 5.6), which was used for the transformation of mulberry seedlings. Mulberry seeds of Guisang You 62 were surface sterilized and sown on MS medium in plastic pots and cultured in an artificial growth chamber at 25 °C. About 2 weeks after germination, the seedlings were selected for agro-infiltration. Firstly, the roots of the mulberry seedlings were cut off, and then the seedlings were immersed in the A. rhizogenes K599 strain suspension for 5 min. Then, the seedlings were cultured on MS medium under dark for 2 d, transferred to MS medium containing 50 g∙L^−1^ kanamycin and 100 g∙L^−1^ terramycin, and cultured for 20 d under light for 16 h per day. After about 4 weeks, mulberry seedlings carrying well-developed hairy roots were used for subsequent experiments.

### 4.6. Plant Treatment

Arabidopsis and mulberry seedlings carrying transgenic hairy roots were grown in plastic pots filled with vermiculite. For the treatment of plant hormones, five-week-old mulberry seedlings were sprayed with 100 μmol∙L^−1^ of ABA, and then mulberry leaves were sampled 0, 2, 4, 6, 8, 10, 12, and 24 h after treatment and stored in a −80 °C freezer after snap-freezing with liquid nitrogen. For salt stress treatment, four-week-old Arabidopsis and five-week-old mulberry seedlings carrying transgenic hairy roots were irrigated with 200 mmol·L^−1^ NaCl solution once. As for the salt stress treatment of leaf discs, transient transformation mulberry leaves were cut into 1.5 cm-diameter discs, which were cultured in a Petri dish with filter paper impregnated with 0 mmol·L^−1^, 200 mmol·L^−1^, 300 mmol·L^−1^, and 400 mmol·L^−1^ NaCl solution, respectively. 

### 4.7. Determination of Na^+^ and K^+^ Contents

Leaves and roots were collected from WT and transgenic plants and dried at 80 °C for 48 h, and then the dried samples were digested with HNO_3_ overnight. The contents of Na^+^ and K^+^ were determined with atomic absorption spectrophotometry (Z-8000; Hitachi, Tokyo, Japan) as described before [45].

### 4.8. Determination of Chlorophylls

Fresh leaf samples (0.5 g) were weighed into a mortar; 10 mL of 80% acetone was added, and they were ground into a homogenate. Then, an appropriate amount of 80% acetone was added again, and the homogenate was transferred to a centrifuge tube. Finally, it was fixed to 20 mL with 80% acetone. Then, the mixture was centrifuged (7500× *g*) for 10 min, and the absorbance of the supernatant was measured at 663 nm, 646 nm, and 470 nm using a UV-Vis spectrophotometer (UV-2100, Beifen-Ruili Analytical Instrument (Group) Co., Ltd., Beijing, China), and the contents of chlorophyll a and b were calculated referring to the methods described before [46]. The experiments were repeated three times.

### 4.9. Measurements of Malondialdehyde and Proline

For the measurement of malondialdehyde (MDA) content, fresh leaf samples were homogenized in trichloroacetic acid (5% *w*/*v*), after which the mixture was centrifuged (10,000× *g*) for 10 min. Then, the supernatant was collected and incubated together with 0.5% thiobarbituric acid in a water bath (100 °C) for 25 min. Then, the mixture was cooled in an ice bath and centrifuged (7500× *g*) for 5 min. After that, the supernatant was collected and used for MDA concentration measurement according to the method described by Liu et al. [47]. For the measurement of proline content, fresh leaf material (500 mg) was extracted with 5 mL of 3% sulfosalicylic acid at 100 °C for 10 min with shaking. The extracts were filtered, and the collected filtrate was used for the analysis of proline content using the acid ninhydrin method [48]. Briefly, 2 mL of the aqueous extract was mixed with 2 mL of glacial acetic acid and 2 mL of acid ninhydrin reagent (1.25 g of ninhydrin, 30 mL of glacial acetic acid, and 20 mL of 6 mol·L^−1^ orthophosphoric acid) and heated at 100 °C for 30 min. After cooling, the reaction mix was partitioned against toluene (4 mL), and the absorbance of the organic phase was determined at 520 nm. The resulting values were compared with a standard curve constructed using known amounts of proline (Sigma, St Louis, MO, USA).

## 5. Conclusions

In conclusion, the *MulSOS2* gene was cloned from mulberry, and our data showed that MulSOS2 protein shares some conserved characteristics with its homologs. Moreover, it was shown that *MulSOS2* was differentially expressed in roots, stems, and leaves, and its expression was induced by NaCl and ABA. In addition, our data showed that the heterogeneous expression of *MulSOS2* in Arabidopsis or its overexpression in mulberry leaves or hairy roots enhanced the salt stress tolerance of the transgenic plants. Furthermore, it was found that *MulSOS2* plays a pivotal function in enhancing osmotic adjustment capacity and reducing oxidative damage to the cell membrane under salt stress. The information provided in this study is helpful for further elucidating the function of the *MulSOS2* gene and providing a basis for improving salt the tolerance of mulberry by genetic engineering and breeding.

## Data Availability

Data are contained within the article.
